# Label Accuracy of Weight Loss Dietary Supplements Marketed Online With Military Discounts

**DOI:** 10.1001/jamanetworkopen.2024.9131

**Published:** 2024-05-01

**Authors:** Cindy Crawford, Bharathi Avula, Andrea T. Lindsey, Kumar Katragunta, Ikhlas A. Khan, Patricia A. Deuster

**Affiliations:** 1 Consortium for Health and Military Performance, Department of Military and Emergency Medicine, F. Edward Hébert School of Medicine, Uniformed Services University, Bethesda, Maryland; 2 Henry M. Jackson Foundation for the Advancement of Military Medicine, Inc, Bethesda, Maryland; 3 National Center for Natural Products Research, School of Pharmacy, University of Mississippi, University, Mississippi

## Abstract

**Importance:**

Dietary supplements for weight loss, among the most popular supplement products on the market, are promoted not only for losing weight and shedding fat, but also for added benefits of energy and performance, all packed into 1 capsule with multiple combinations of ingredients. Fraudulent marketing of weight loss supplements, some with exaggerated claims, some that are potentially dangerous, and some that contain illegal ingredients, is ever present, especially through online sources, where multiple manufacturers target service members by offering military discounts.

**Objectives:**

To examine whether select dietary supplements marketed online for weight loss from companies advertising military discounts are accurately labeled according to the Supplement Facts listed ingredients, whether they contain any ingredients prohibited for use in the military, and to qualitatively describe the products’ label claims.

**Design, Setting, and Participants:**

In this case series, 30 dietary supplement products marketed for weight loss were selected and purchased in June 2023 from 12 online companies advertising military discounts. Data were analyzed from July to August 2023.

**Main Outcomes and Measures:**

Liquid chromatography-mass spectrometry was used to verify whether products were accurately labeled according to the Supplement Facts listed ingredients and whether they contained any substances on the DoD Prohibited Dietary Supplement Ingredients List. A separate analysis was conducted to describe product label claims by using the Operation Supplement Safety (OPSS) Risk Assessment Scorecard.

**Results:**

Of the 30 products tested, analysis showed that 25 had inaccurate labels. Of these, 24 had ingredients listed on the label that were not detected (misbranded); 7 had hidden components not present on the label, some of which would be considered adulterated; and 10 had substances on the DoD Prohibited Dietary Supplement Ingredients List either on or hidden from the label. All products were rated as risky when applying the OPSS Scorecard.

**Conclusions and Relevance:**

In this case series study, the majority of products had inaccurate labels. Some were misbranded, others would be considered adulterated with ingredients not allowed in dietary supplements, and some contained ingredients prohibited for use in the military.

## Key Points

**Question:**

Are select dietary supplements marketed for weight loss that offer military discounts accurately labeled according to the Supplement Facts listed ingredients on product labels?

**Findings:**

This case series study analyzed 30 dietary supplement products purchased from online companies advertising military discounts for products with claims about weight loss. Twenty-five had inaccurate labels, 24 were misbranded, 7 had hidden components detected, and 10 contained substances prohibited for military use.

**Meaning:**

These findings suggest that predatory marketing and low quality of weight loss supplements pose a threat to military members and the public.

## Teaser

This case series study evaluates whether dietary supplements marketed online for weight loss from companies advertising military discounts are accurately labeled.

## Introduction

The global market for weight loss dietary supplements is estimated at US $43.9 billion in 2022 and projected to rise to US $135.7 billion by 2030.^1^ Losing body weight is one of the top reasons for dietary supplement use.^2,3^ The demand for these supplements is ever-present as people attempt to take control of their own health and pursue healthy lifestyles, now more than ever.^4,5^ The growing obesity concern is one factor associated with the demand for these products. However, the advertising market has also shifted toward more holistic solutions offered by weight loss products, with the added benefit of not only losing weight and shedding fat, but also building lean muscle and enhancing energy and performance. Many different ingredients are packed into one pill with various combinations to include vitamins, minerals, herbal extracts, and purportedly natural stimulants. This strategy is likely to rapidly expand the growth opportunities for the market^6^; with service members a prime target for the industry. In fact, surveys have shown that service members use more dietary supplements than civilians and that their use of these combination products has increased in recent years.^7,8,9^

Misinformation surrounding dietary supplements is common as individuals often obtain information through social media channels, peer-to-peer communication, and other online sources, which are not necessarily evidence-based or trustworthy for making informed decisions.^10,11^ According to a Council for Responsible Nutrition report, most Americans believe the dietary supplement industry is trustworthy (77% of all US adults and 84% of supplement users) and 85% are confident in the safety and quality of supplements overall.^12,13,14^ People tend to mistakenly believe that dietary supplements have been declared safe and effective by the Food and Drug Administration (FDA) if they are available for purchase in stores and online, despite a body of research showing that weight loss products are often misbranded, contaminated, or even adulterated with illegal ingredients.^15,16,17,18,19,20^ These dietary supplement products have been associated with adverse events such as stroke, hepatoxicity, and even death.^20,21,22,23,24,25,26,27^ The most recent statistics from 2004 to 2013 showed that dietary supplements were associated with an estimated 23 000 emergency department visits each year and many were due to products marketed as weight loss.^28^ It is unknown what these statistics are today. In addition, the FDA hosts a Weight Loss Tainted Products database, and has identified over 100 weight loss dietary supplements containing hidden ingredients, such as blood pressure medications, seizure medications, and/or drugs not approved in the US.^29^ Unfortunately, the FDA cannot test all weight loss products that enter the market, so the products identified by the FDA likely constitute only a small fraction of the potentially dangerous products available to consumers.

Service members may face serious consequences beyond exposure to a potential serious adverse health effect or a resulting performance decrement, such as the risk of positive drug tests, which could jeopardize their military careers if they mistakenly take a product that contains a drug (prescription or unapproved) or other substance prohibited for use.^26,30^ Yet, fraudulent marketing of weight loss products—some with exaggerated claims, some that are potentially dangerous, and some containing illegal ingredients—continues, especially through online sources. Some sources even target service members through offering military discounts. Service members need to know that the weight loss products they access through online sources are of high quality.

The purpose of this case series study was to analyze and qualitatively describe select weight loss dietary supplement products being marketed online from companies specifically advertising military discounts. Funding allowed for the selection of 30 products in total. Products were tested to assess quality and verify whether they were accurately labeled according to the Supplement Facts listed ingredients and identify any prohibited ingredients contained in products according to the DoD Prohibited Dietary Supplement Ingredients List (DoD Prohibited List).^31^ The Operation Supplement Safety (OPSS) Risk Assessment Scorecard criteria^32,33^ was used as a screening tool to assess a product’s relative safety potential and to mitigate risk based solely upon the product label claims.

## Methods

Thirty dietary supplement products were selected and purchased over the internet for this case series with claims about weight loss from various online shops that advertise military discounts for dietary supplements in June, 2023. First, the primary author (C.C.) signed out of Google and cleared cache, cookies, and browser history. From the Chrome browser, “weight loss supplements military discount” was entered into Google search engine. Next, hyperlinks that appeared on the results page, contained the text of “military discount,” and led directly to a company website selling dietary supplement products were recorded in the order of first appearance. The author selected up to 5 weight loss products from each company’s website (going down the list of results), until a total of 30 products were identified for purchase from 12 distinct companies. If a specific company had more than 5 weight loss products available for purchase, the author filtered the products advertised as best sellers, highest ratings, or narrowed the search to a goal within weight loss (fat burning) if available. The author did not examine the claims or Supplement Facts labels as part of the selection of products. In accordance with the Common Rule, the authors did not seek institutional review board approval since this case series evaluated products, not patients. The reporting guideline for case series and the Strengthening the Reporting of Observational Studies in Epidemiology (STROBE) reporting guideline were followed, as applicable to case series.^34^

One sample of each selected product was purchased over the internet and sent to the University of Mississippi’s National Center for Natural Products Research for product analysis. Liquid chromatography-mass spectrometry was used to determine the quality of the 30 dietary supplement products.^19,35,36^ All ingredients listed on the label and any hidden compounds were analyzed except for lipids and minerals. The list of ingredients detected through analysis for each product was compared with the ingredients on the product’s Supplement Facts label to determine whether the product’s label was accurate. Full methods are detailed in the eMethods in Supplement 1.

### Data Analysis

A separate qualitative analysis was conducted by the authors not affiliated with University of Mississippi by using the set of questions from the OPSS Scorecard^33^ in parallel with the testing (see eMethods in Supplement 1). This scorecard was developed as an educational tool to help consumers learn about a product by carefully reading the label and quickly assessing whether it might be risky based solely upon the label claims.^32,36^ Authors read the labels of the products and answered 7 questions about topics such as third-party certification and proprietary blends. A score of 4 or more is “likely okay/less risky;” less than 4 is considered a “no-go/risky.” If a product contained any ingredients on the DoD Prohibited List,^31^ it was also a “no-go.” All analyses were conducted in Excel 2019 (Microsoft) in duplicate by 2 authors (A.T.L. and C.C.). Any discrepancies were resolved through discussion and consensus. Data were analyzed from July to August 2023.

## Results

### Product Analysis

Twenty-five of the 30 products (83%) had inaccurate labels confirmed through product testing. Twenty-four (80%) had ingredients listed on the label that were not detected through analysis, such that their labels were misbranded. Ingredients not detected from products ranged from 1 (5%) to 4 (44%) missing from any single product. Not detected ingredients included plant extracts such as bitter orange whole plant, hoodia Chinese extract, *Huperzia serrata* extract, kola nut, raspberry ketones, *Rauwolfia*, and white willow bark extract. Seven products (23%) contained additional hidden components detected that were not present on the label. Hidden compounds consisted of 1,4 DMAA, butyrylcarnitine, epigallocatechin gallate (EGCG), methylsynephrine (oxilofrine), *N*-methyl-β-phenylethylamine, *N*-phenethyl dimethyl amine (*N, N*-DMPEA), and yohimbine. One product claimed saffron but *Carthamus tinctorius* flower was detected instead, a less expensive substitute for saffron. Six products (20%) were both mislabeled and had hidden components detected as not present on the label (Table).

**Table.** Product Analysis of 30 Weight Loss Dietary Supplements Tested

Product No.	No. of ingredients presented on product label	Product label verification accuracy	Ingredients on label analyzable but not detected, No. (%)^a^	Additional or hidden components detected not present on the label	DoD prohibited ingredients detected	WADA S6 stimulants detected
1	14	Accurate	None	None	None	None
2	14	Not accurate	1 (8)	None	None	None
3	20	Not accurate	3 (20)	None	None	None
4	15	Not accurate	3 (21)	None	Hordenine Hydrochloride;^b^ Vinpocetine;^b^ Higenamine Hydrochloride;^b^ 2-Aminoisoheptane Hydrochloride (DMHA)^b^	None
5	10	Not accurate	1 (10)	None	Octopamine Hydrochloride;^b^ 1,4 DMAA^b^	N-Phenethyl dimethyl amine (N,N-DMPEA); β-Phenylethylamine Hydrochloride (β-PEA)
6	12	Accurate	None	None	Isopropylnorsynephrine^b^	None
7	11	Not accurate	1 (10)	None	None	None
8	14	Not accurate	None	Butyrylcarnitine	Hordenine Hydrochloride^b^	None
9	12	Not accurate	3 (25)	Yohimbine	None	None
10	15	Not accurate	2 (14)	None	None	None
11	11	Accurate	None	None	Higenamine Hydrochloride;^b^ Hordenine Hydrochloride;^b^ N-Isopropylnorsynephrine^b^	N-Phenethyl dimethyl amine (N,N-DMPEA)
12	29	Not accurate	1 (5)	None	None	None
13	19	Not accurate	2 (11)	None	Higenamine Hydrochloride;^b^ Isopropylnorsynephrine^b^	β-phenylethylamine (β-PEA)
14	18	Not accurate	1 (7)	None	None	None
15	9	Accurate	None	None	None	None
16	14	Not accurate	1 (9)	None	None	N-Phenethyl dimethyl amine (N,N-DMPEA)
17	8	Not accurate	1 (13)	None	None	None
18	21	Not accurate	1 (5)	None	None	None
19	10	Not accurate	1 (11)	*Carthamus tinctorius* flower	None	None
20	27	Not accurate	4 (15)	None	None	None
21	8	Not accurate	1 (13)	None	None	None
22	15	Not accurate	1 (7)	1,4 DMAA	Ephedrine, methylephedrine and pseudoephedrine;^b^ Methylsynephrine;^b^ 2-Aminoisoheptane Hydrochloride (DMHA);^b^ 1,4 DMAA^c^	Phenylethylamine (β-PEA); N-phenethyl dimethylamine (N,N-DMPEA); N-methyl-β-phenylethlamine
23	9	Not accurate	2 (22)	EGCG	7-[2-hydroxy-3-(2- hydroxyethyl- methylamino)propyl]- 1,3-dimethylpurine-2,6- dione^b^	2-Phenylethan-1-amine
24	11	Not accurate	1 (9)	1,4 DMAA	2-Aminoisoheptane Hydrochloride (DMHA);^b^ ephedrine, methylephedrine and pseudoephedrine;^b^ Methylsynephrine;^b^ Higenamine Hydrochloride;^b^ 1,4 DMAA^c^	Phenylethylamine (β-PEA); N-phenethyl dimethylamine (N,N-DMPEA);* N*-methyl-β-phenylethlamine
25	13	Not accurate	3 (30)	None	None	None
26	14	Not accurate	3 (21)	*N* -methyl-β-phenylethylamine Methylsynephrine (Oxilofrine) *N*-Phenethyl dimethyl amine (*N, N*-DMPEA)	Ephedrine and pseudoephedrine, methylephedrine;^b^ *Acacia rigidula;*^b^ Methylsynephrine (Oxilofrine)^c^	*N*-methyl-β-phenylethylamine; *N*-Phenethyl dimethyl amine (*N, N*-DMPEA)
27	10	Accurate	None	None	None	None
28	10	Not accurate	1 (10)	None	None	None
29	10	Not accurate	4 (44)	None	None	Phenylethylamine Hydrochloride
30	18	Not accurate	1 (6)	None	None	None

^a^ The number of analyzable ingredients differs from the number of ingredients presented on the product label due to the instrumentation limits for analysis. Elements including zinc, selenium, calcium, chromium, manganese, magnesium, potassium, sodium, and so forth are not able to be analyzed as well as fat soluble vitamins, including Vitamin D3.

^b^ Ingredient appeared on label.

^c^ Ingredient did not appear on label.

Ten of the 30 products (30%) contained ingredients listed on the DoD Prohibited List^31^ either on or hidden from the label. Prohibited ingredients confirmed through product testing ranged from 1 to 7 from any single product and consisted of the following: 2-aminoisoheptane HCl (DMHA), 1,4 DMAA, *Acacia rigidula*, ephedra extract listed on labels but detected as ephedrine, methylephedrine and pseudoephedrine, higenamine, hordenine, isopropylnorsynephrine, methylsynephrine, octopamine, and vinpocetine. An unapproved drug, 7-[2-hydroxy-3-(2-hydroxyethyl-methylamino)propyl]-1,3-dimethylpurine-2,6-dione, known as Xanthinol, was listed on a label and detected in 1 product.^37^

Nine products contained stimulants of phenethylamine and its derivatives, prohibited in sport according to the World Anti-Doping Agency^38^ (WADA) including: β-phenylethylamine HCl (β-PEA), *N*-phenethyl dimethyl amine (*N, N*-DMPEA, *N, N* -dimethylphenylethylamine), and *N*-methyl-β-phenethylamine. Seven products had ingredients on both the DoD Prohibited List and the WADA List (S6 stimulants), with 2 to 10 of these ingredients in any one of the 7 products.

### Product Descriptions

The 12 online stores offered military discounts for up to 20% off dietary supplement products, with language such as, “we’re proud to offer...to salute military personnel,” “we offer discounts as a way to say thanks,” and “to thank you for your service.” The 30 weight loss products not only had claims about “crushing” weight loss and shedding fat, but additional claims for enhanced energy, mood, and mental clarity all in one pill. Examples of claims made on product bottles were: “shed fat while building lean muscle,” “also gives you energy, heightened mood,” “combat mental fatigue,” “destroy body fat,” “without the jitters,” and “fat incinerating best friend.” Nineteen of the products also had additional scientific-sounding claims such as, “clinically effective,” “clinically proven,” “scientifically formulated,” “super-dosed, potent combination,” “maximum strength,” and “scientifically researched and proven ingredients.” One product was advertised as made specifically for the “elite athlete.” The cost of these products ranged from $19.99 to $59.99 with a median (IQR) cost of $39.99 ($14.10) for a 30-day supply.

No product had an independent, third-party certification seal on the label to show that it had been tested by an independent laboratory for quality and/or contaminants, such as BSCG Certified Drug Free, NSF Certified Sport, LGC’s Informed Sport, or USP (United States Pharmacopeia). Fourteen of the products had a Good Manufacturing Practice (GMP) seal or GMP text on their product label. GMP is an FDA requirement, but such seals should not be mistaken as indications of safety, quality, or federal approval. Still other products had seals stating “tested for purity” or “lab tested,” but not associated with any known independent, third-party organization.

The number of ingredients present on the Supplement Facts labels ranged from 8 to 29 (mean [SD], 14.03 [5.18]) on any single product. Fourteen products consisted of proprietary blends, matrices, or complexes, where the consumer is not privy to the amount of each ingredient(s) contained in the proprietary blend, thus the actual content of the entire product was unclear. Twenty-one of the 30 products listed caffeine on Supplement Facts labels as either: (a) contained in a proprietary blend (where it was not possible to know the amount) or (b) in amounts over 200 mg per serving. Besides caffeine, which was contained in all products, other common ingredients listed on labels included green tea extract (17 products), yohimbe/yohimbine (15 products), black pepper (13 products), L-tyrosine (12 products), grains of paradise (6-paradol) (12 products), acetyl-L-carnitine (10 products), huperzine A (10 products), *Citrus aurantium* [standardized for *p*-synephrine] or synephrine (10 products), *Rauwolfia*/rauwolscine (8 products), and raspberry ketones (7 products). In addition, all products were rated as “no-go/risky” according to the OPSS Scorecard.

## Discussion

In this study, we analyzed 30 weight loss dietary supplement products advertised with military discounts online. Our analyses revealed that 25 of the 30 products tested for quality had inaccurate labels: 24 had ingredients listed on the label but not detected in the product (misbranded), 7 had hidden components not present on the label, and 10 contained ingredients on the DoD Prohibited List, either on the label or hidden from the label. All products were rated as risky according to the OPSS Scorecard. In addition, no product was third-party certified to show that the product had been tested and verified for its quality to mitigate risk.^39^

The predatory marketing to service members and low quality of dietary supplements promoted for weight loss pose a threat to military members and the public. Phrases such as, “We’re proud to offer …,” “… to salute our Service Members,” “to help you take your fitness to the next level,” and “to empower and support our military,” are of concern because these products might actually threaten the health, readiness, performance, and careers of those who volunteer to defend our country. Beyond the dangers to one’s health and career, the cost of these products may create financial burdens on individuals, often without a positive return on investment, and may contribute to a financial readiness issue for military families. Overall, the findings from this study are problematic and require solutions. As of now, the only way to know the actual ingredients in a product is to ensure it has been tested by an independent third-party organization.

Of concern was the fact that most of the products contained multiple ingredients, and multiple combinations with stimulant effects. It is unknown how these stimulant and other ingredients interact with each other, not to mention current use of over-the-counter and/or prescription medications or additional dietary supplement products. Also, some products listed herbal/botanical extracts containing compounds such as methylsynephrine, *N*-methyl-β-phenylethylamine, *N,N*-dimethylphenylethylamine, where the plant authenticity could not be verified. In fact, adulteration of botanicals to boost profits has occurred with unintentional or intentional substitution or dilution with undisclosed lower-cost ingredients.^40,41^ These issues present clear health risks for all consumers.

Of note, the DoD has taken a number of actions to protect personnel from problematic ingredients that appear in marketed supplements.^42^ For example, in 2002, the DoD banned the sale of products containing ephedrine alkaloids from military commissaries worldwide. The FDA banned ephedra in April 2004. In 2011, after many adverse events and several deaths were linked with the use of products containing a synthetic ingredient, DMAA, the DoD restricted its sale on bases. Although the FDA banned the ingredient in April 2013, our analyses reveal products still contain both DMAA and ephedra alkaloids, with the ingredients sometimes still listed right on the labels.

In January 2012, the Assistant Secretary for Health Affairs requested a DoD-wide educational campaign on dietary supplements, OPSS, to increase awareness within the DoD community about potential health risks and how to choose safe dietary supplements.^43^ Due to the frequency of dietary supplement use by service members, an overall DoD policy was developed as guidance to ensure safe dietary supplement use and minimize risks to the force. In 2022, the DoD issued Instruction 6130.06, Use of Dietary Supplements in the DoD, in which the DoD establishes OPSS as the go-to program for dietary supplements.^44^ It states that OPSS will maintain the official DoD Prohibited List, and provide educational training on the topic of dietary supplements for all service members and those who provide health-related services to the military. OPSS provides the tools and resources to help users make informed decisions about dietary supplements and actively educates on this topic with health care providers, allied health professionals, and all service members. More education as well as other solutions are needed to address this public health issue. It’s imperative that our service members remain healthy and ready to serve, and not be put in harm’s way with predatory marketing.

### Limitations

This analysis has limitations worth noting. First, these 30 products are not meant to be representative of all weight loss dietary supplement products found online, nor do they represent all product advertisements targeted toward service members with military discounts. Second, fat soluble vitamins including Vitamin A/D/E/K, lipids (oils), and elements cannot be detected and/or have low sensitivity using liquid chromatography-mass spectrometry, and thus those ingredients listed on product labels were not analyzable. In addition, the analysis was qualitative in nature in that the amount of each ingredient detected was outside the scope of the project and thus the authors cannot comment on whether the amount detected of any ingredient matches the labeled amount for any one ingredient.

## Conclusions

In summary, our analysis does suggest that predatory marketing and access to low-quality dietary supplements is present through online sources. The majority of products analyzed had inaccurate labels, some were misbranded, some would be considered adulterated with ingredients not allowed in dietary supplements, and some contained ingredients prohibited for use in the military.

## Notes

Supplement 1.
eMethods.

eReferences.

Supplement 2.
Data Sharing Statement
