# Supplementary material for: Label Accuracy of Weight Loss Dietary Supplements Marketed Online With Military Discounts
Source: JAMA Netw Open. 2024 May 1;7(5):e249131. doi: 10.1001/jamanetworkopen.2024.9131 (PMC11063798; doi:10.1001/jamanetworkopen.2024.9131)
Supplement: Supplement 1. — eMethods. eReferences. [file jamanetwopen-e249131-s001.pdf]

Supplemental Online Content

Crawford C, Avula B, Lindsey AT, Katragunta K, Khan IA, Deuster PA. Label accuracy of weight loss dietary supplements marketed online with military discounts. *JAMA Netw Open*. 2024;7(5):e249131. doi:10.1001/jamanetworkopen.2024.9131

eMethods

eReferences

This supplemental material has been provided by the authors to give readers additional information about their work.

© 2024 Crawford C et al. *JAMA Network Open.*

eMethods:

Analytical Methods

1. Chemicals and reagents

HPLC grade acetonitrile, methanol and formic acid were purchased from Fisher Scientific (Fair Lawn, NJ, USA). Water was obtained using a milliQ-Gradient system.

2. Preparation of dietary supplement samples

The dietary supplements were encountered in the form of either capsules, tablets, or powders. For capsules, 5 items were weighed, opened and their contents were mixed and triturated in a mortar and pestle prior. Each dietary supplement purchased as powders or capsules or tablets, about 1000 mg for powders and average weight in case of capsule content or tablets were weighed into centrifuge tubes, re-suspended with methanol, vortex and sonicated for 30 minutes, following centrifugation for 15 minutes at 959 x g. The procedure was repeated for three times and the clear supernatant was subsequently transferred to a 10 mL volumetric flask. The final volume was adjusted with methanol to 10 mL and mixed thoroughly. Prior to injection, the samples were filtered through a 0.45µm polytetrafluoroethylene (PTFE) membrane filter.

3. Instrumentation

Liquid Chromatography-Quadrupole Time-of-Flight Mass Spectrometry (LC-QToF-MS)

The analytical methodology is same as reported elsewhere. [1-3] The liquid chromatographic system is an Agilent Series 1290 and the mass spectrometric analysis was performed with a QToF-MS/MS (Model #G6530A, Agilent Technologies, Palo Alto, CA, USA) equipped with an ESI source with Jet Stream technology. All the operations, acquisition and analysis of data were controlled by Agilent MassHunter Acquisition Software Ver. A.10.00 and operated under MassHunter Workstation software Ver. B.10.00. Each sample was analyzed in both positive and negative modes to provide abundant information for structural identification. Mass spectra are recorded across the range *m/z* = 50-1700 with accurate mass measurement of all mass peaks. MassHunter Workstation software, including Qualitative Analysis (version B.10.00), was used for processing both raw MS and MS-MS data, including molecular feature extraction, background subtraction, data filtering, and molecular formula estimation. The raw data were processed using the Find by Molecular Feature (MF) algorithm called Molecular Feature Extractor (MFE) within MassHunter Qualitative Analysis software. Extracted molecular features were processed to create a list of compounds.

A compound search for the non-targeted compounds were characterized by matching the experimental molecular formula in an [a] The Agilent MassHunter Forensics and Toxicology (>9000 components) Personal Compound Database (PCD) [b] In-House generated library for 15,000 components of medicinal plant samples. Other search engines included SciFinder (web-based version), Dictionary of Natural product (CRC, 2023), and google search engines by molecular formulae were used for the identification of "known unknowns." These approaches have been utilized to identify a wide range of components, including additives, compounds from natural products, etc. In-house library includes the compound name, molecular formula, exact mass, CAS #, and structure of each compound. From the possible positive hits, the results were compared with MS-MS experiments and to those available in literature. All compounds either generated a high-abundance $[M-H]^-$ or/and $[M+HCOO]^-$ ion in negative mode or a high-abundance $[M+H]^+$ or/and $[M+Na]^+$ ion in positive mode, therefore, the $[M-H]^-$ or $[M+H]^+$ ions of each compound were selected as the precursor ions for subsequent MS-MS experiments to give more fragment ions. The generation of diagnostic fragment ions provided information concerning the core skeleton and nature of the substituents and helps in identification of the compounds.

Operation Supplement Safety (OPSS) Risk Assessment Scorecard[4]

The OPSS Scorecard was developed as an educational tool to help consumers learn about a dietary supplement product by carefully reading the label and quickly assess whether it might be risky based solely upon the label claims. There are seven yes/no questions in total as detailed below. A score of four or more yes's is "likely okay/less risky," whereas less than four is considered a "no-go/risky." In addition, if the product contains any ingredients on the *DoD Prohibited Dietary Supplement Ingredients List*, it is a "no-go." An interactive version of the Scorecard can be accessed here: https://www.opss.org/opss-scorecard-check-your-dietary-supplement.

1. Is any one of these third-party certification seals on the product label? (BSCG Certified Drug Free, NSF Certified Sport, LGC's Informed Sport, or USP (United States Pharmacopeia)
2. Are there less than six ingredients on the Supplement Facts label?
3. Is the label free of the words proprietary, blend, matrix, or complex?
4. Can you easily pronounce the name of each ingredient on the Supplement Facts label?
5. Is the amount of caffeine listed on the label 200 mg or less per serving? (If caffeine is not listed, mark "Yes")

© 2024 Crawford C et al. *JAMA Network Open.*

6. Is the label free of questionable claims or statements?
7. Are all the % Daily Values (%DV) on the Supplement Facts label less than 200%? (If % DV is not listed, mark “No”)

eReferences

1. Avula B, Chittiboyina A, Bae J, et al. The power of hyphenated chromatography-time of flight mass spectrometry for unequivocal identification of spirostanes in bodybuilding dietary supplements. *J Pharm Biomed Anal.* 2019;167:74-82.
2. Crawford C, Avula B, Lindsey AT, et al. Analysis of select dietary supplement products marketed to support or boost the immune system. *JAMA Netw Open.* 2022;5(8):e2226040.
3. Crawford C, Walter AR, Avula B, et al. Relative safety and quality of various dietary supplement products U.S. Service Members ask about. *Clin Toxicol (Phila).* 2022;60(6):737-744.
4. Operation Supplement Safety's Scorecard: Check your dietary supplement. https://www.opss.org/opss-scorecard-check-your-dietary-supplement.

© 2024 Crawford C et al. *JAMA Network Open.*
