# Supplementary material for: Label Accuracy of Weight Loss Dietary Supplements Marketed Online With Military Discounts
Source: JAMA Netw Open. 2024 May 1;7(5):e249131. doi: 10.1001/jamanetworkopen.2024.9131 (PMC11063798; doi:10.1001/jamanetworkopen.2024.9131)
Supplement: Supplement 2. — Data Sharing Statement [file jamanetwopen-e249131-s002.pdf]

Data Sharing Statement

Crawford. Label Accuracy of Weight Loss Dietary Supplements Marketed Online With Military Discounts. *JAMA Netw Open*. Published May 01, 2024.
doi:10.1001/jamanetworkopen.2024.9131

Data
Data available: Yes
Data types: Data (not involving human participants)
How to access data: cindy.crawford.ctr@usuhs.edu
When available: With publication

Supporting Documents
Document types: None

Additional Information
Who can access the data: Anyone requesting the data
Types of analyses: for any purpose
Mechanisms of data availability: with a signed data access agreement
Any additional restrictions: Data are kept within an Excel worksheet and can be shared; not relevant to this study
